# Dose-Dependent Phytotoxic Mechanism of UV-328 on the Photosynthetic System of the Moss *Niphotrichum japonicum* (Dozy & Molk.) Bedn.-Ochyra & Ochyra

**DOI:** 10.3390/plants15152248

**Published:** 2026-07-23

**Authors:** Xiaoqing Zhang, Fei Xu, Mingyan Yang, Wen Ye

**Affiliations:** 1 Modern Industrial College of Traditional Chinese Medicine and Health, Lishui University, Lishui 323000, China; yokyzhang@163.com (X.Z.); xfddhx@163.com (F.X.); 2 College of Agriculture and Biotechnology, Lishui University, Lishui 323000, China; 18386893365@163.com; 3 School of Life Sciences, Xiamen University, Xiamen 361102, China

**Keywords:** UV-328 stress, moss, phototoxicity, OJIP, JIP test

## Abstract

First detected in environmental matrices in the early 2010s, UV-328 has aroused widespread concern due to its toxic risks to terrestrial vascular plants. However, its impacts on photosynthetic performance of bryophytes remain unclear. In this study, we exposed the moss *Niphotrichum japonicum* to a range of UV-328 concentrations and examined its morphological changes, oxidative stress, photosynthetic pigments, chlorophyll fluorescence kinetics, and energy allocation. Our results show that UV-328 caused dose-dependent leaf yellowing, surface shrinkage, and papillae collapse. It degraded photosynthetic pigments, disrupted chlorophyll a/b balance, and induced excess reactive oxygen accumulation. Chlorophyll fluorescence data indicated that UV-328 noticeably lowered Fv/Fm, Y(II), and qP. OJIP kinetics confirmed multi-target damage to the photosynthetic chain, including disruption of the oxygen evolving complex, decline of PSII energetic grouping, blockage of electron transfer from QA^−^ to QB, inhibition of plastoquinone pool turnover, and PSI terminal electron transport. Energy flux parameters pointed to fewer active PSII reaction centers and a higher light-harvesting load on each remaining center, which uncoupled light capture from electron transfer. The concentration that caused clear photosynthetic damage fell between 100 and 150 μM. In conclusion, UV-328 impacts moss photosynthesis through oxidative stress, structural damage, blocked electron transport, and energy imbalance.

## 1. Introduction

Benzotriazole UV stabilizers (BUVs), one of the most concerned emerging contaminants, have been frequently found in global water environments [1], air [2], dust [3], soil [4], and living organisms [5]. They are widely utilized in industrial sectors such as plastics, coatings, automotive components, aerospace materials, and textiles [6,7], as well as in personal care products like sunscreens and liquid foundations [8,9]. UV-328 (2-(2-hydroxy-4′,6′-di-tert-amylphenyl) benzotriazole), one of the most extensively utilized UV stabilizers, is widely applied to alleviate photodegradation of plastic products caused by solar UV radiation [10]. Owing to its environmental persistence, bioaccumulation, and potential toxicity, the European Commission revised the EU Regulation on Persistent Organic Pollutants (POPs) in 2025. The revision added UV-328 to the control list and explicitly banned its production, use, and trade, making it the first non-halogenated persistent organic pollutant subjected to global regulatory control [11,12].

Existing monitoring data indicate that UV-328 concentrations across diverse environmental matrices span several orders of magnitude, exhibiting pronounced regional and matrix-specific variations [13,14]. In surface waters, UV-328 has been detected at concentrations ranging from 2.8 to 287 ng/L [13]. Sediments serve as a major environmental sink for UV-328, where concentrations have been reported at 0.72 to 224 ng/g dw [15]. Notably, BUVs concentrations in air and dust are substantially higher, reaching μg/g dw levels in certain indoor environments, ranging from 689 to 11,023 ng/g dw [16].

UV-328 was first detected in wildlife, including fish and birds, and has since been found in mammals and humans. Cadena-Aizaga et al. (2022) studies on coastal fish in the Canary Islands revealed UV-328 concentrations of 1.34–45.6 ng/g dw in fish tissues [17]. Its presence in breast milk, fat tissue, and urine points to widespread exposure [18,19,20]. This wide distribution has raised serious concerns about risks to both ecosystems and human health. Emerging evidence suggests that UV-328 not only interferes with the endocrine system, but also exhibits reproductive, developmental, neurotoxic, and immunotoxic effects [21,22]. In zebrafish, UV-328 causes abnormal heart rates and increases the expression of inflammatory cytokines such as IL-12 and TNF-α [23]. Furthermore, it triggers excessive production of reactive oxygen species (ROS) through the MAPK signaling pathway, leading to oxidative stress [23,24,25].

In terms of phytotoxicity, BUVs can enter agricultural ecosystems by wastewater irrigation, plastic film degradation and atmospheric deposition. BUVs exhibit pronounced plant-specific bioaccumulation, with root concentration factors ranging from 47.9 to 464 mL/g in lettuce and 194–787 mL/g in radish, and preferential aerial tissue accumulation in reeds [26,27]. They significantly affect plant (such as rice, lettuce, pakchoi and radish) growth and development, physiological metabolism, and molecular regulation [28,29,30,31]. Studies have found that BUVs in protected agricultural soils with greenhouses and plastic mulching reach up to 68.2 ng/g [4,32]. These findings raise serious concerns regarding widespread exposure risks to crops and humans.

In rice, UV-328 turns down more than half of the genes needed for chlorophyll production while turning up those linked to chlorophyll degradation. Meanwhile, BUVs speed up glycolysis and the TCA cycle, which consumes small carbohydrates. They also shift the flow of carbohydrates and amino acids, and the plant’s antioxidant defense becomes weaker [33]. Furthermore, other BUVs have been found to interfere with the ABA signaling pathway and stomatal closure in rice by competitively binding to calcium-dependent protein kinases (CDPKs) [34]. The effects of BUVs on plant growth depend on concentration and can be biphasic. A volume of 50 μM UV-328 promotes the growth and photosynthetic performance of *Arabidopsis thaliana*. In contrast, 150 μM causes chloroplast swelling, damages thylakoid structure, and lowers the activity of genes involved in light harvesting [28].

In the aquatic environment, BUVs are toxic to green algae. UV-328 sharply raises ROS levels in algal cells and acts at both molecular and cellular levels to trigger oxidative stress [35]. When the green alga *Chlamydomonas reinhardtii* was exposed to UV-328 for 96 h, lipid peroxidation went up noticeably, and antioxidant defenses like glutathione peroxidase (GPX) were activated [35]. Although numerous studies have investigated the effects of UV-328 on plant growth and physiological processes, most have focused on algae and a limited number of plant species, with an emphasis on photosynthesis or oxidative toxicity in organisms. Consequently, risk assessments for other ecosystems remain largely lacking.

Bryophytes, with simple morphological structure, large specific surface area, and heightened sensitivity to environmental changes, are widely recognized as bioindicators of pollution [36,37,38]. These plants can efficiently adsorb and sequester contaminants directly by their epidermal surfaces, and specialized structures in certain species further enhance this sequestration capacity [39,40]. Under stressful conditions, bryophytes exhibit overt phenotypic symptoms such as chlorophyll catabolism and cellular structural damage, which subsequently impair photosynthetic functionality, metabolic homeostasis, and the stability of gene expression profiles [28]. Compared with vascular plants, bryophytes react faster and more directly to pollutants, which makes them well-suited for studying how plants respond to environmental stress.

*Niphotrichum japonicum* (Dozy and Molk.) Bedn.-Ochyra and Ochyra, usually known by its basionym *Racomitrium japonicum* Dozy and Molk., belongs to the family Grimmiaceae. As a representative species across east Asia, *N. japonicum* occurs in various habitats, especially harsh spots like dry rocks under strong sunlight. Owing to its remarkable tolerance to heat, drought, intense light, and low nutrient availability [41,42,43], this species has been widely used in urban greening projects, particularly on rooftops and walls [43]. Beyond landscaping, *N. japonicum* also serves as a pollution indicator [44,45]. Several studies have looked at how it resists stress, focusing on photosynthesis, physical changes, and molecular pathways [46,47]. Meanwhile, research on Antarctic bryophytes has revealed special ways they cope with high UV radiation, such as making UV-blocking compounds and turning on ROS scavenging systems [48].

With this in mind, we used *N. japonicum* to see how UV-328 stress affects its photosynthesis and antioxidant responses. Our results should help clarify how UV-328 harms plants, support more realistic assessments of its ecological risk in farmlands and provide a useful basis for managing the use and safety of benzotriazole-based UV stabilizers.

## 2. Results

### 2.1. Dose-Dependent Response of UV-328 on N. japonicum Morphology and Cell Structure

**Figure 1:**
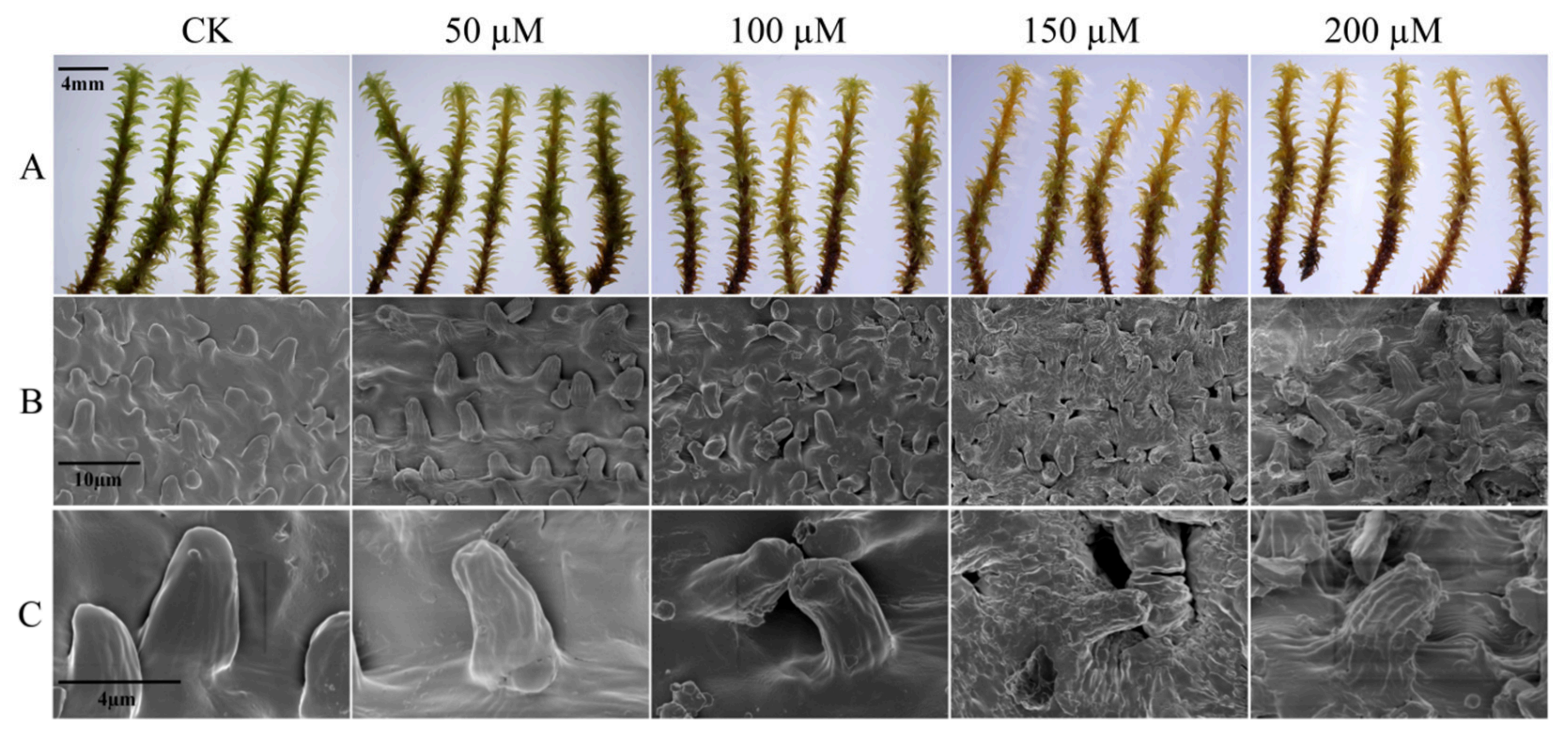
Morphological alterations (**A**) and microstructural changes (**B**,**C**) in *N. japonicum* under UV-328 stress. The SEM images of leave upper surface (**B**) at 10 µm and papillae (**C**) at 4 µm.

The growth characteristics of *N. japonicum* were analyzed after a range of UV-328 concentration treatments. Notably, the moss morphology exhibited a distinct dose-dependent tendency. As shown in Figure 1A, the leaf color changes from green to yellow with the treatment concentration increasing. Regarding the leaf micro-structure, scanning electron microscopy (SEM) observations of the leaf surface morphology revealed that with increasing concentrations, the leaf surface transitioned from smooth to wrinkled, and the papillae became disordered and irregular in arrangement (Figure 1B). Compared to the smooth surface of the control group, the papillae also exhibited varying degrees of shrinkage in morphology. The papillae under 150 and 200 µM UV-328 treatment displayed severe collapse and rupture (Figure 1C).

### 2.2. Effect of UV-328 Stress on Chlorophyll Content and ROS Accumulation

**Figure 2:**
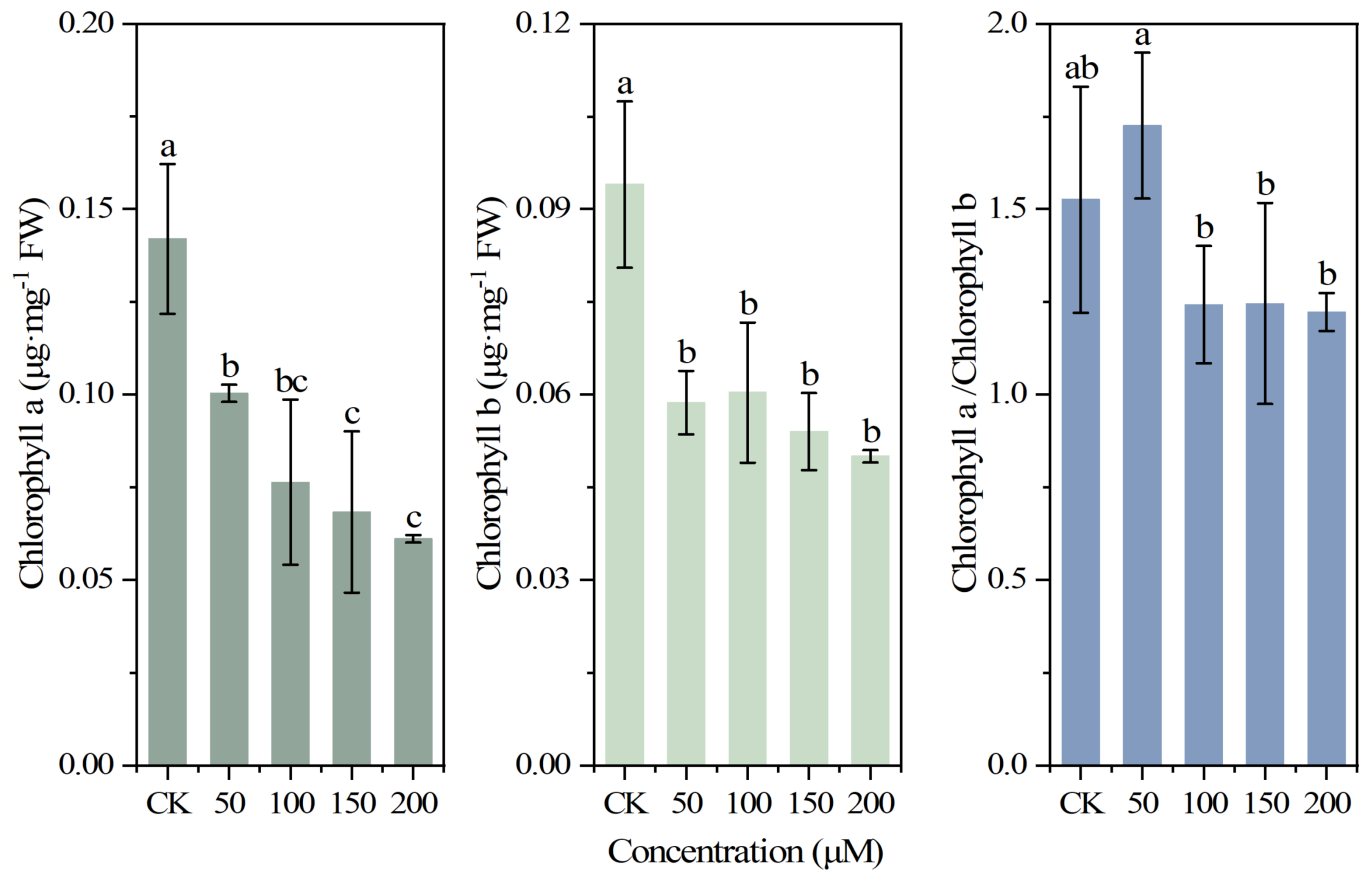
Chlorophyll content of *N. japonicum* after UV−328 stress. Different letters represent the significant differences between treatments at *p* ≤ 0.05. Values are mean ± SD (*n* = 3).

Previous studies have shown that ultraviolet radiation can turn down key genes that code for chlorophyll a and b binding proteins [49]. Therefore, changes in chlorophyll content may act as an effective indicator for assessing photosynthetic damage of *N. japonicum*. As shown in Figure 2, UV-328 exposure resulted in a significant dose-dependent decrease in chlorophyll content. Compared with the control group, treatment with 50 μM UV-328 decreased chl a and chl b by 29.37% and 37.59%, respectively. At 100 μM, chl a and chl b were significantly reduced by 46.27% and 35.82%. With a further increase in concentration, chl a and chl b decreased markedly, declining by 51.90% and 42.55% at 150 μM, and by 57.04% and 46.81% at 200 μM. Correspondingly, the chl a/b ratio increased by 13.07% at 50 μM but gradually declined with increasing UV-328 concentration, with no significant differences among groups.

**Figure 3:**
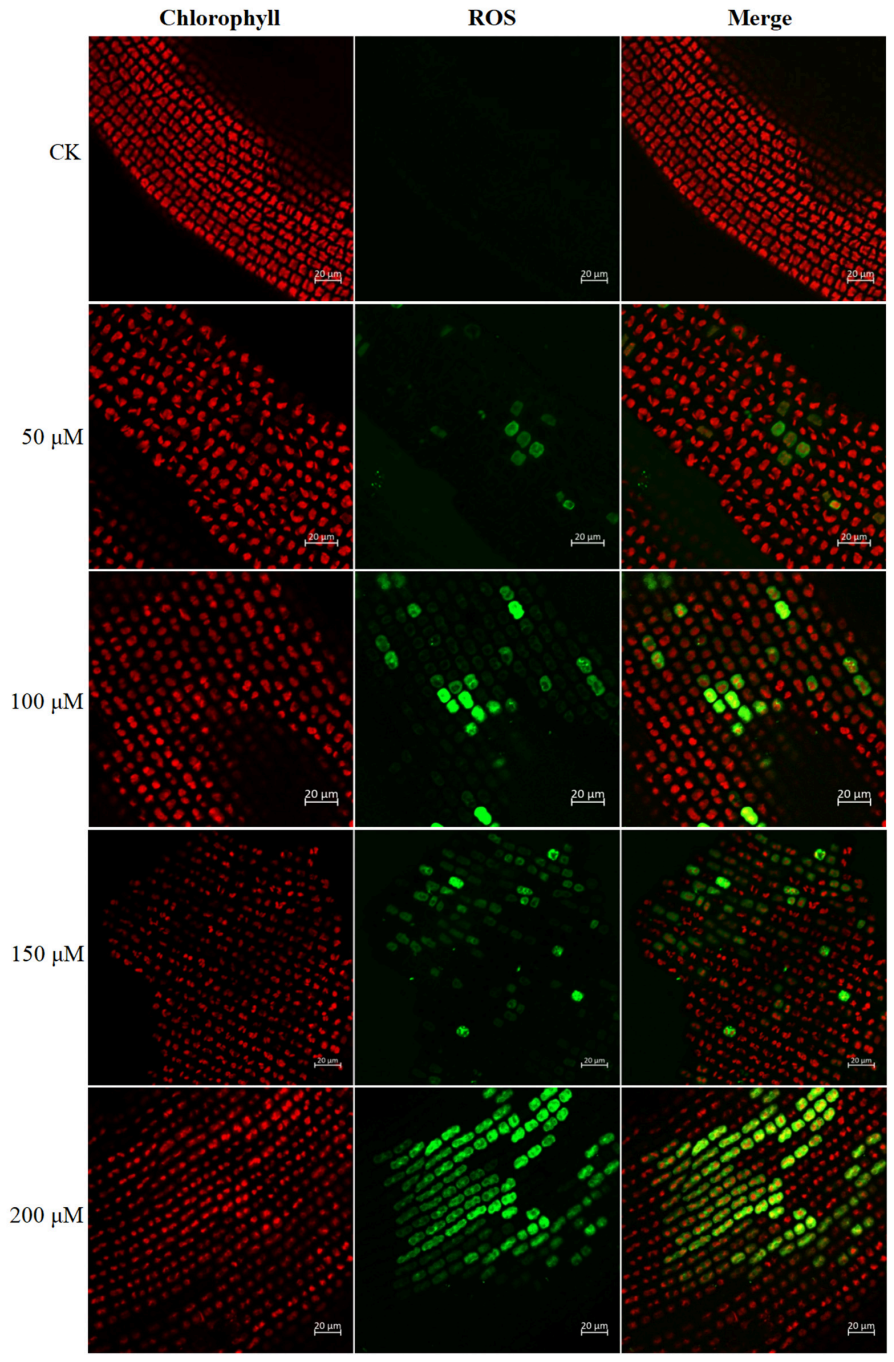
Confocal laser scanning microscopy (CLSM) images of *N. japonicum* phylloids under UV-328 stress with dichlorofluorescein (DFC)-labeling. The I column shows the chlorophyll autofluorescence, the II reports DCF signal and the III is the merge.

As shown in Figure 3, confocal microscopy examinations of DCF-labeled leaves showed green fluorescent signals from ROS in the cytoplasm and red autofluorescent signals from chlorophyll. The results revealed that the effects of UV-328 stress on chloroplasts and ROS in *N. japonicum* cells were dose-dependent. In the control group, the autofluorescence of chlorophyll was uniform and dense, with almost no detectable green ROS fluorescence. In the 100 μM group, the quantity and intensity of ROS fluorescence increased significantly. However, chlorophyll fluorescence remained dense and no obvious damage to chloroplast structure was observed. When the concentration was increased to 150 μM, the density of chlorophyll fluorescence decreased slightly, accompanied by expanded distribution range and continued elevation of ROS accumulation. In the 200 μM group, ROS fluorescence was highly expressed in patches, which extensively overlapped with chlorophyll fluorescence, and the integrity of chloroplast structure was more significantly damaged.

### 2.3. Chlorophyll Fluorescence Imaging of the N. japonicum Under UV-328 Stress

**Figure 4:**
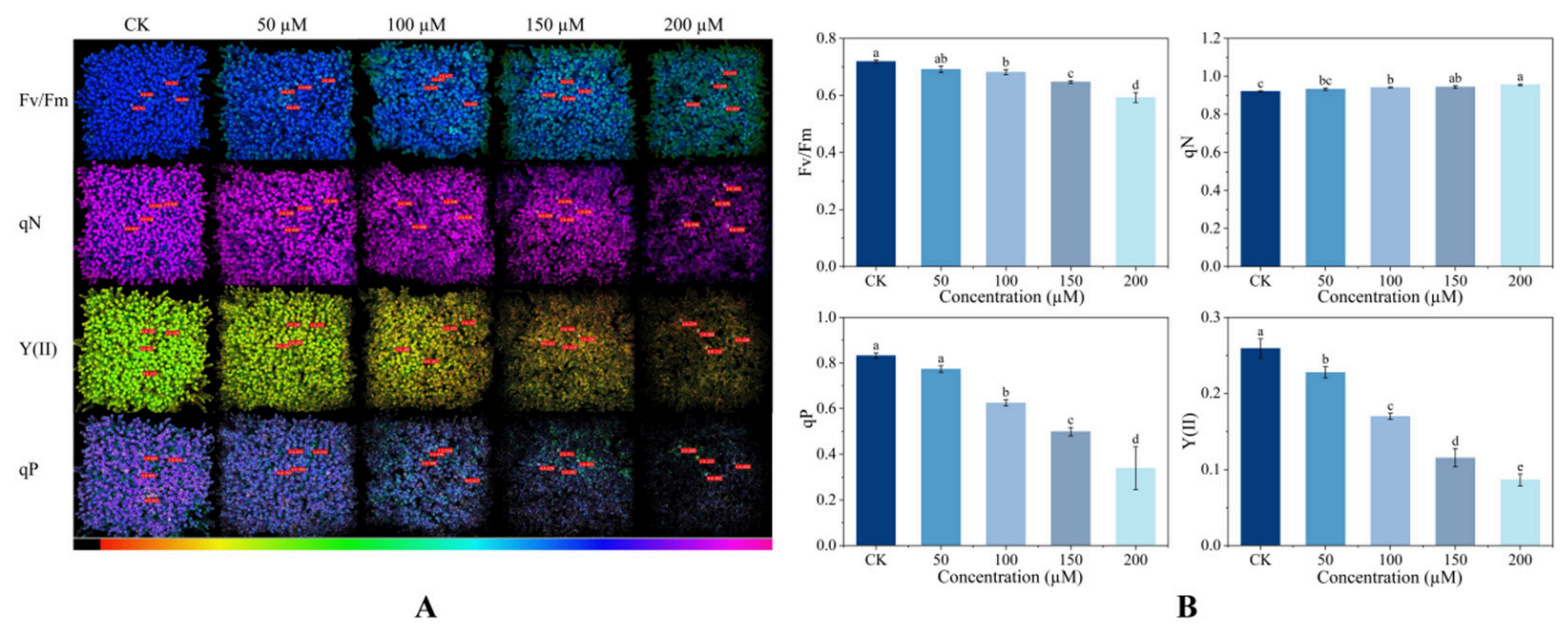
Chlorophyll fluorescence imaging (**A**) and parameters (**B**) of *N. japonicum* under different UV-328 concentrations. The false color code depicted at the bottom of each image ranges from 0.000 (black) to 1.000 (pink). Different letters represent the significant differences between treatments at *p* ≤ 0.05. Values are mean ± SD (*n* = 3).

Chlorophyll fluorescence parameters act as intrinsic indicators for exploring how plant photosynthesis responds to the environment. Therefore, we determined chlorophyll fluorescence imaging (Figure 4A) and chlorophyll fluorescence parameters (Figure 4B) of *N. japonicum*, in order to evaluate the impact of UV-328 stress on its photosynthetic performance. As one of the most common parameters, Fv/Fm represents the PSII quantum yield and reflects the plant’s light energy conversion ability. As UV-328 concentrations went up, Fv/Fm in *N. japonicum* gradually dropped. At 50 and 100 µM, it fell by 3.86% and 5.25%, respectively. At the medium dose of 150 µM, the drop became more pronounced, reaching 9.99%. When the concentration reached 200 µM, Fv/Fm dropped sharply by 17.61%. As a marker of actual PSII photochemical efficiency, Y(II) accurately reflects the light energy conversion capacity of the photosynthetic system. When *N. japonicum* was exposed to UV-328, Y(II) went down with increasing concentrations. Compared with the control, the decreases were 12.19% at 50 μM, 34.5% at 100 μM, 55.45% at 150 μM, and a sharp 66.61% at 200 μM. During photosynthesis, fluorescence quenching splits into photochemical quenching (qP) and non-photochemical quenching (qN), which reflect the intensity of photosynthetic activity and thermal dissipation, respectively. Compared with the control, qP dropped markedly with rising concentrations. It went down by 7.1% at 50 µM, 24.9% at 100 µM, 40.11% at 150 µM, and 55.3% at 200 µM. In contrast, qN showed a slight rise across all concentrations. Even in 200 µM group, qN increased by only 3.79%, with no statistically significant difference observed.

### 2.4. Chlorophyll a Fluorescence Rise Kinetics of N. japonicum Under UV-328 Stress

The OJIP kinetics and the JIP-test, being highly sensitive and information-rich tools, have been extensively used for monitoring changes in the photosynthetic apparatus under various stress conditions [50].

#### 2.4.1. Chlorophyll Fluorescence Induction Kinetics Raw Curve and Relative Variable Fluorescence V_t_

**Figure 5:**
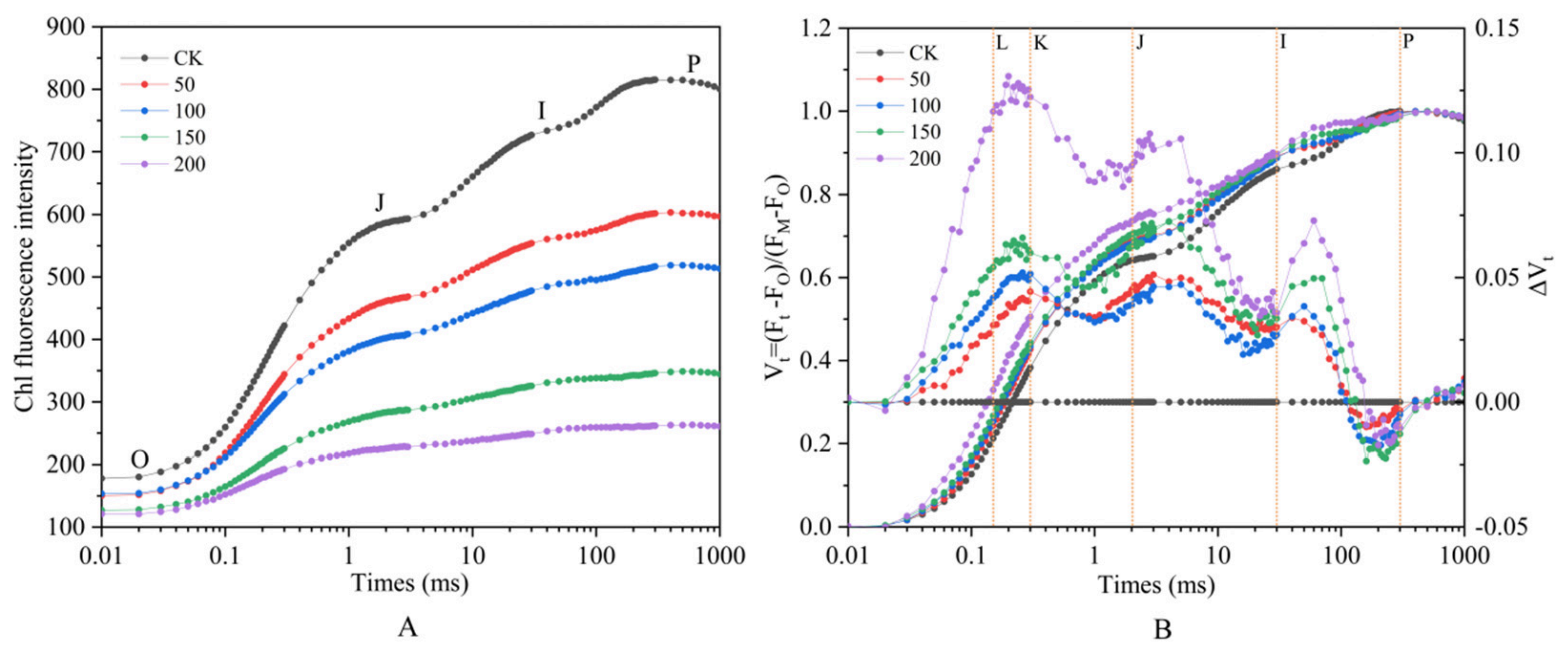
Chlorophyll fast-phase fluorescence of *N. japonicum* under different UV−328 concentrations. (**A**) Raw Chl a fluorescence rise kinetics on a logarithmic time scale. (**B**) Chl a fluorescence rise kinetics normalized by F_O_ and F_M_ as V_t_ = (F_t_ − F_O_)/(F_M_ − F_O_) in a logarithmic time scale. ΔV_t_ = V_t_ (treatment) − V_t_ (control). Each value of point in curve represents the average of 30 measurements.

As shown in Figure 5A, the OJIP curves of *N. japonicum* treated with UV-328 were presented. Compared with the control, as UV-328 concentration increased, initial fluorescence (F_O_) gradually decreased, and maximum fluorescence (F_M_) significantly dropped, particularly in the 200 μM. At higher treatment concentrations (150 and 200 μM), the OJIP multi-phase transient curve of *N. japonicum* almost disappeared and became linear.

To gain more detail than raw OJIP curves can offer, the OJIP curves were double-normalized between F_O_ and F_M_ to obtain relative variable fluorescence V_t_ and ΔV_t_ at a logarithmic time scale. As shown in Figure 5B, with the UV-328 concentration increasing, the O, J, and I point on the OJIP curve all moved higher. The rise at the J point suggests that electron transport on the acceptor side of PSII was held back. During the O-J phase, there was a clear increase, which was followed by the emergence of the K point. Meanwhile, the P point dropped steadily with higher concentrations and ended up below the control level.

Analysis of ΔV_t_ revealed that treatments varied noticeably during the O-J phase (Figure 5B). As the stress concentration increased, the ΔV_t_ near the L, K, and J points became more pronounced. At 200 μM, the ΔV_t_ of this treatment differed significantly from the others. In contrast, differences near the I and P points were small, and the values at the P point fell below the control, resulting in negative values.

#### 2.4.2. The O-K Phase

**Figure 6:**
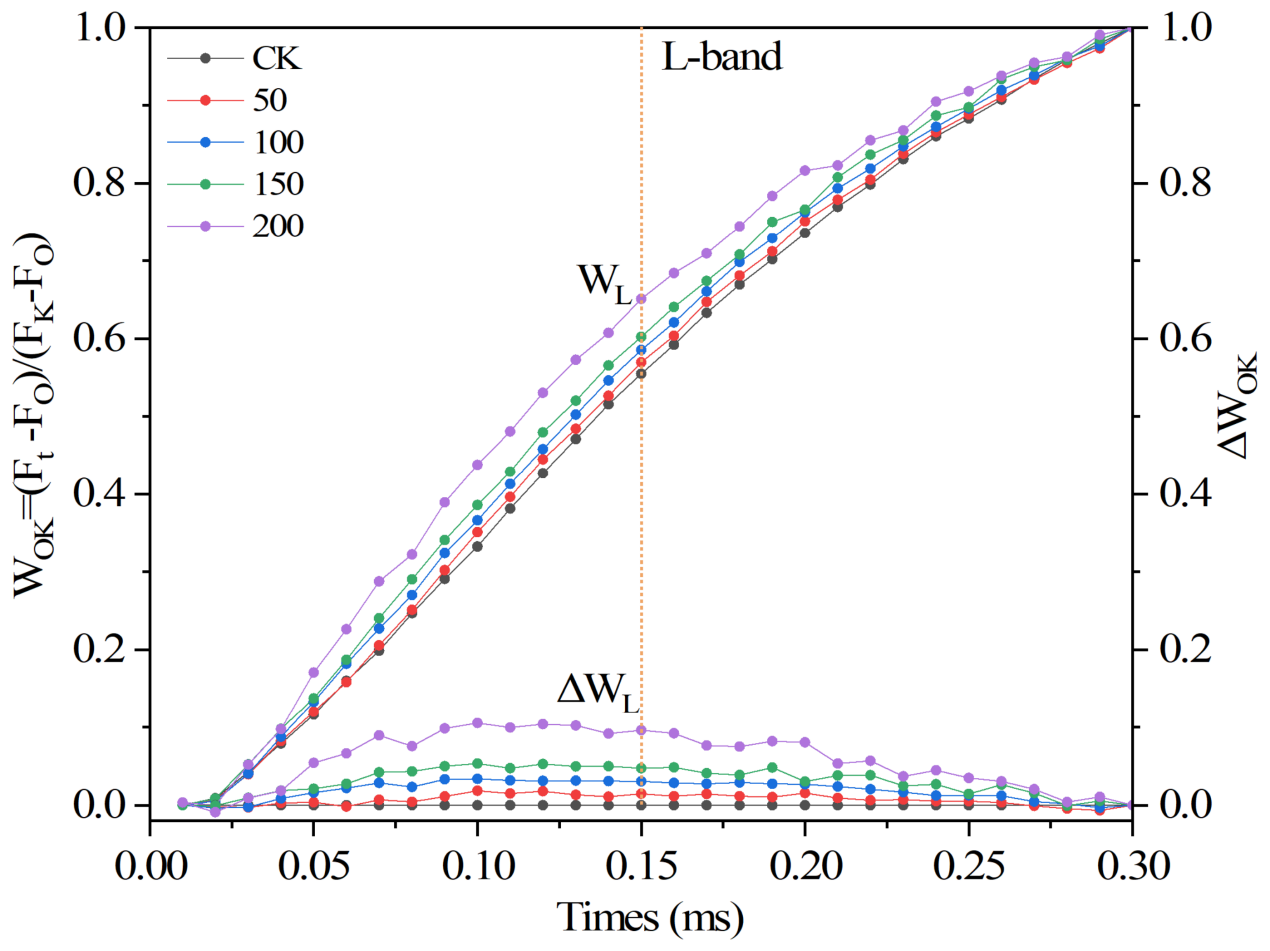
Fluorescence rise kinetics normalized by F_O_ and F_K_ as W_OK_ = (F_t_ − F_O_)/(F_K_ − F_O_), and the difference kinetics ΔW_OK_ = W_OK_ (treatment) − W_OK_ (control) in a linear time scale from 0 to 300 μs. The ratio of relative variable fluorescence at the L-step to the amplitude F_K_ − F_O_ as W_L_ = (F_150μs_ − F_O_)/(F_K_ − F_O_) and the difference in W_L_ as ΔW_L_ = W_L_ (treatment) − W_L_ (control).

To better understand the details in the O-K phase, the OJIP curves were double-normalized by F_O_ and F_K_ to visualize the L peak as W_OK_ = (F_t_ − F_O_)/(F_K_ − F_O_) and then plotted with the difference kinetics ΔW_OK_ = W_OK_ (treatment) − W_OK_ (control) in the linear time scale from 0 to 300 μs (Figure 6). We observed the L-band at approximately 150 μs, a signal normally hidden within the O-K phase. This band reflects the extent of energy coupling and clustering among PSII units. Lower connectivity or grouping probability makes the L-band more distinct [51]. Across all groups, W_OK_ increased progressively during the O-K phase, consistent with the standard fluorescence rise from F_O_ to the K-step. No significant difference between the treatments and the control was observed at the early stage (0–0.05 ms). With time, the W_OK_ difference between treated groups and the control widened gradually in a dose-dependent manner. At 0.15 ms, the 200 μM group showed a notably higher W_OK_ than the other groups. At the L-band time point, ΔW_L_ was positive in all treatments and significantly larger in high-dose than low-dose groups, which indicating that UV-328 reduces energy transfer efficiency among PSII reaction centers in a dose-dependent manner.

#### 2.4.3. The O-J Phase

**Figure 7:**
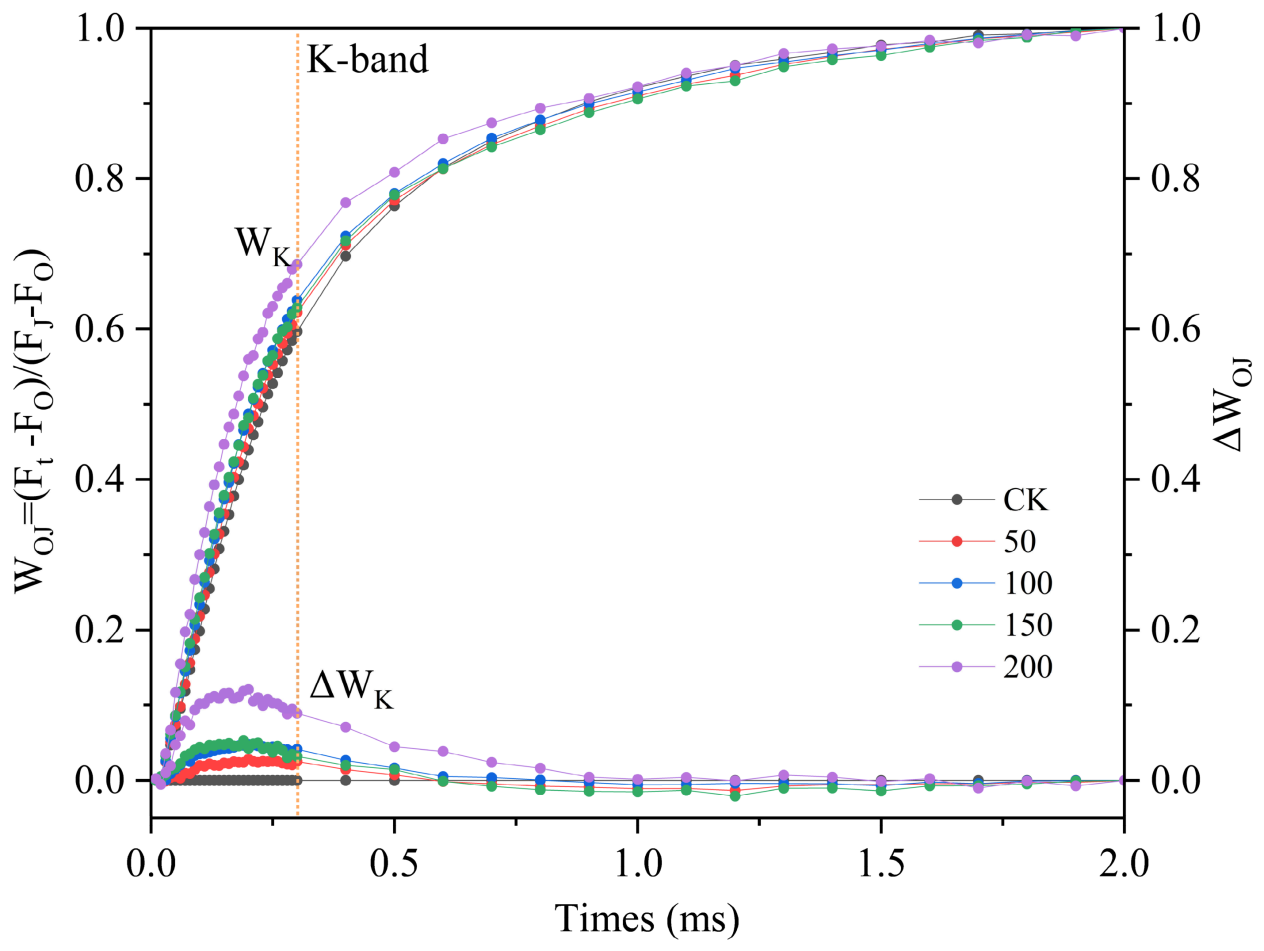
Fluorescence rise kinetics normalized by F_O_ and F_J_ as W_OJ_ = (F_t_ − F_O_)/(F_J_ − F_O_), the difference kinetics ΔW_OJ_ = W_OJ_ (treatment) − W_OJ_ (control) in a linear time scale from 0 to 2 ms.

As shown in Figure 5B, a noticeable positive K peak of ΔW_OJ_ was detected. ΔW_OJ_ in the linear time range of 0 to 2 ms revealed the K peak after the OJIP curves were double-normalized between the O-step (20 μs) and J-step (2 ms). Dose-dependent alterations in fluorescence intensity were observed from the very early stages (0.01–0.3 ms) (Figure 7).

To further clarify the stress response, we analyzed the difference kinetics ΔW_OJ_. It became positive immediately after illumination and increased rapidly in all treatment groups. The 200 μM group reached its first peak at approximately 0.05–0.15 ms. The changes of W_OJ_, specifically the K-band signal in the O-J phase, directly reflect the functional state of the OEC on the PSII donor side. A more pronounced K-band, shown by higher W_OJ_ and a positive ΔW_K_, is widely used as a marker of OEC damage. The dose-dependent increase in W_OJ_ and ΔW_K_ demonstrates that UV-328 disrupts OEC function. As the core component responsible for water splitting and donor-side electron supply, OEC damage restricts electron flow to the PSII reaction center, leading to abnormal fluorescence rise in the O-J phase.

#### 2.4.4. The O-I Phase

The J-I-P transition of the W_OI_ curve reflects the kinetics of electron transfer from the acceptor side of PSII to PSI. The J-I segment is mainly governed by PQ pool turnover, whereas the amplitude of the I-P rise is directly associated with the pool size of PSI terminal acceptors.

**Figure 8:**
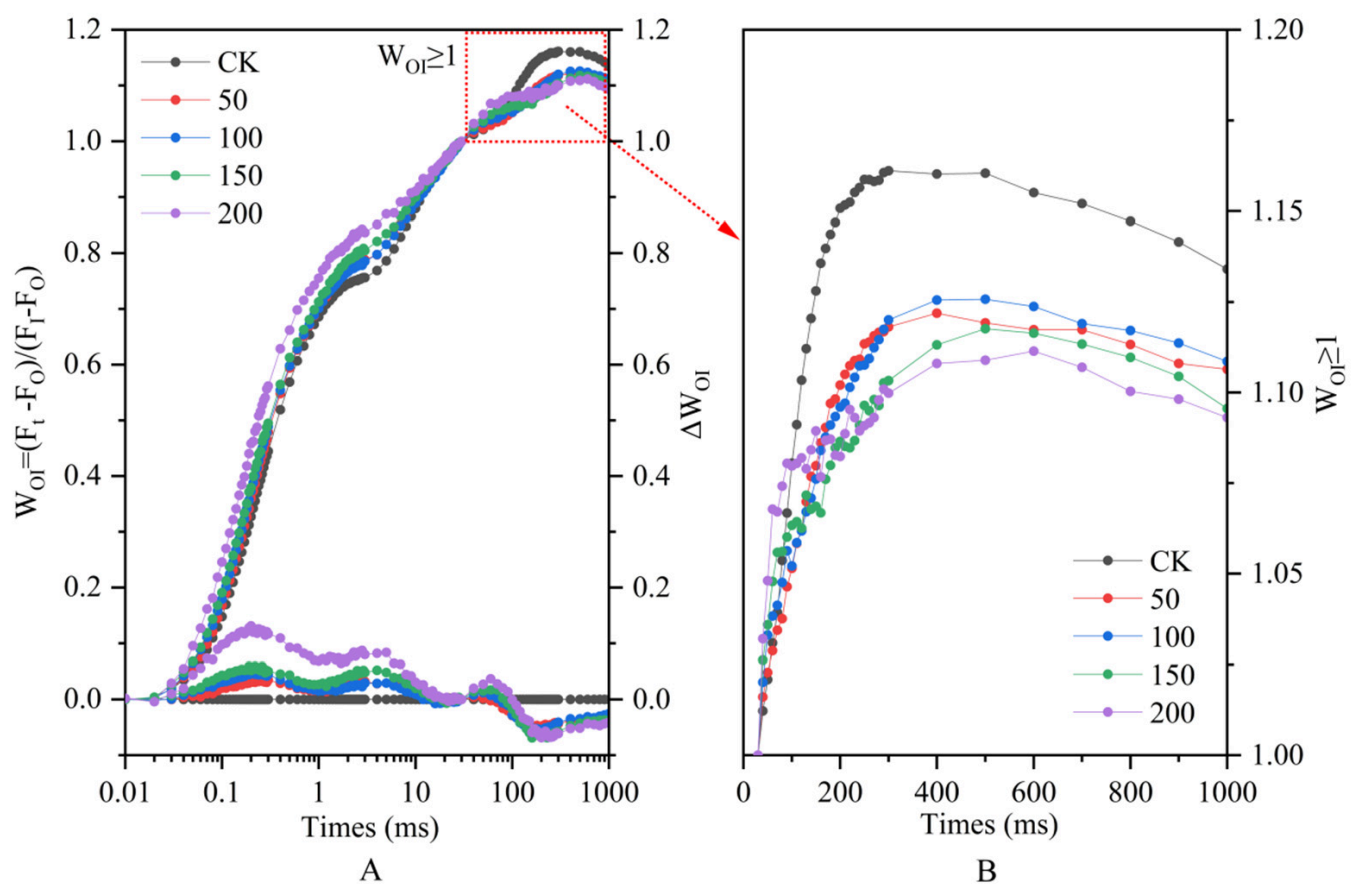
Fluorescence rise kinetics normalized by F_O_ and F_I_ as W_OI_ = (F_t_ − F_O_)/(F_I_ − F_O_), the difference kinetics ΔW_OI_ = W_OI_ (treatment) − W_OI_ (control) (**A**) and W_OI_ ≥ 1 (**B**).

Consistent with analyses of other phases, we further evaluated the effects of different treatments by performing double normalization of the O-I phase on a logarithmic time scale. During the O-J phase, W_OI_ curves were similar to the V_t_ curves, with all treatments exhibiting a typical rapid increase (Figure 8). The J-I phase, associated with PQ pool reduction and electron filling from QA^−^ to the PQ pool, showed a dose-dependent response. At 50–150 μM, ΔW_OI_ dropped below zero following the J point, and this decrease became more distinct with higher doses. These results point to accelerated electron transfer to the PQ pool or enhanced re-oxidation of the pool, which would explain why less relative fluorescence accumulated than in the control. By contrast, ΔW_OI_ declined slowly and remained positive under 200 μM treatment, indicating a relatively reduced PQ pool. In all treatment groups, ΔW_OI_ was negative, indicating restricted electron transport toward PSI acceptors. However, the negative shift at 200 μM was significantly smaller than that observed at lower concentrations. This result implies stronger inhibition of PSI terminal acceptor re-oxidation, over-reduction in the PSI acceptor side, and reduced fluorescence quenching during this phase.

### 2.5. Pipeline Models of Specific Energy Fluxes per PSII Active Reaction Center

**Figure 9:**
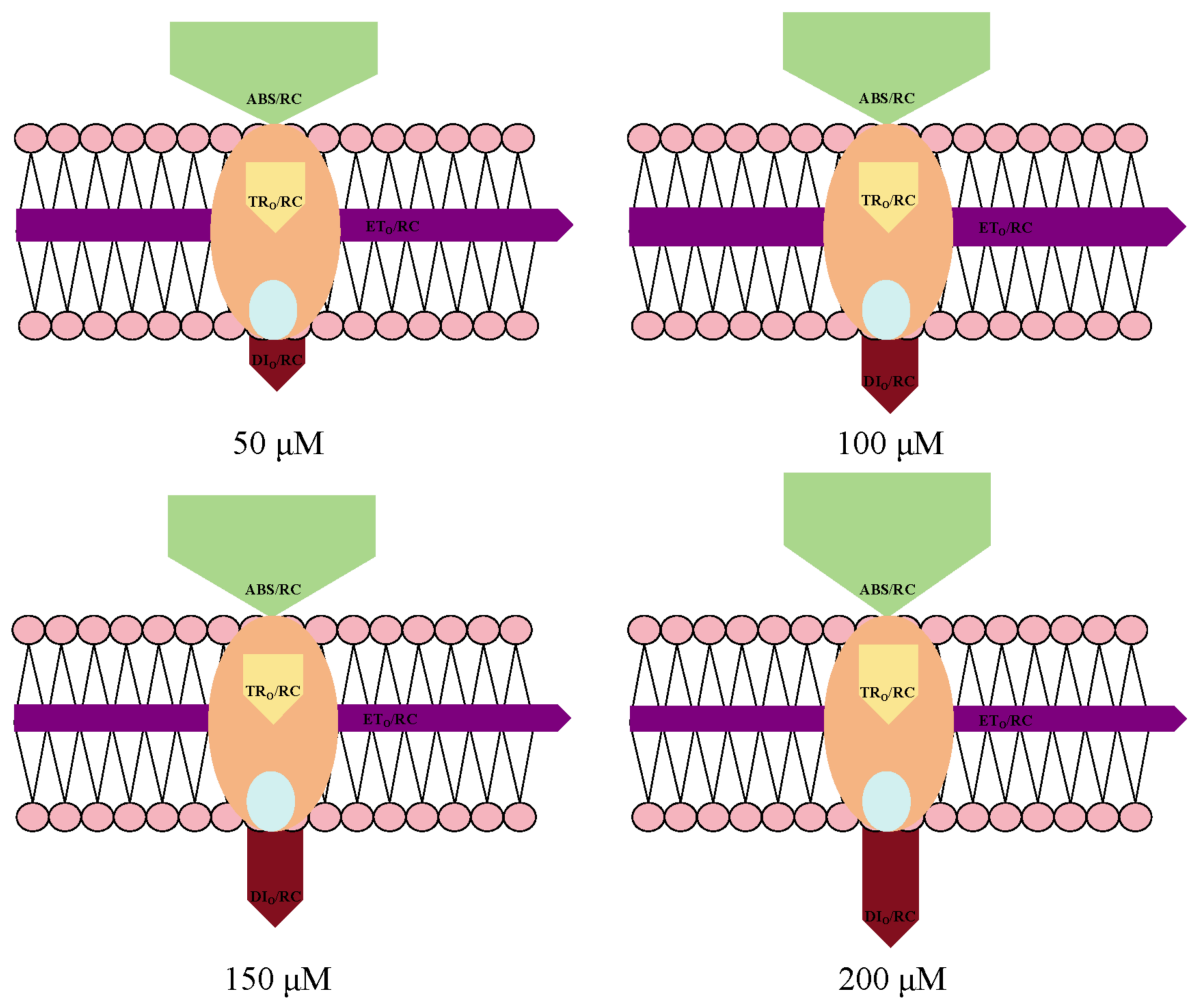
Pipeline models showing per-active-PSII energy fluxes in *N. japonicum* exposed to various UV-328 concentrations. ABS/RC, photon absorption per active PSII; TR_0_/RC, trapped energy per active PSII; DI_0_/RC, energy dissipated (as heat and fluorescence) per active PSII; ET_0_/RC, electron flow from QA^−^ to the PQ pool per active PSII.

We evaluated the impact of UV-328 stress on PSII reaction centers in *N. japonicum* by measuring specific energy fluxes per active reaction center and normalizing them to the control (Figure 9). UV-328 exposure clearly modified energy allocation within PSII. ABS/RC rose in a dose-dependent manner and reached its maximum at 200 μM. This suggests that fewer PSII reaction centers remained active, so each active center had to absorb more light. Meanwhile, DI_0_/RC rose sharply, especially at 200 μM, pointing to greatly enhanced thermal dissipation of excess excitation energy.

TR_0_/RC remained relatively stable across all treatments, indicating that the capacity for excitation energy trapping and QA reduction was preserved. ET_0_/RC, however, decreased substantially with increasing concentration, especially at 150 and 200 μM. The combination of stable TR_0_/RC and reduced ET_0_/RC points to impaired electron transport downstream of QA^−^, including QA^−^ re-oxidation and electron transfer into the PQ pool. Thus, UV-328 stress primarily suppresses PSII photochemical efficiency by blocking electron transfer on the acceptor side. Although reaction centers kept their ability to absorb light and reduce QA, downstream electron flow was restricted, and the excess energy was mostly dissipated as heat.

### 2.6. JIP-Test Analysis

**Figure 10:**
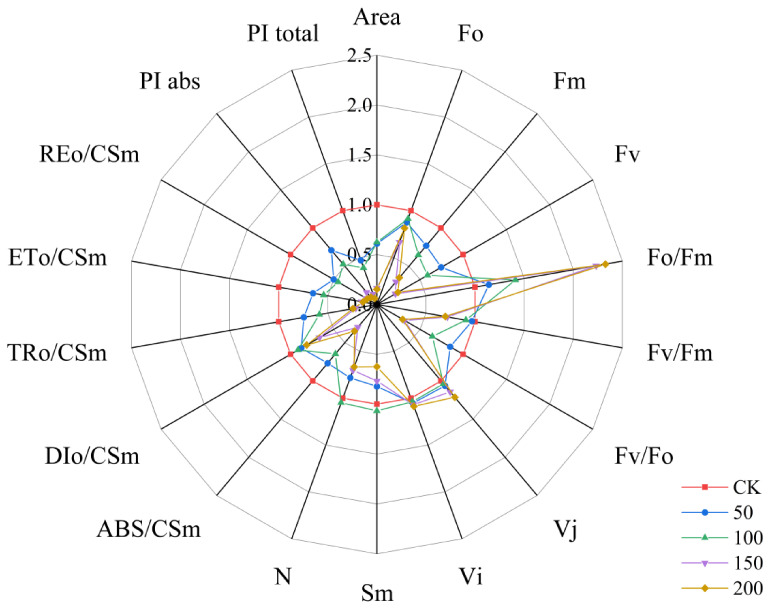
Spider plots of the photosynthetic parameters in different treatments. All the values were normalized to the control as 1.

To further understand the effects of UV-328 stress on the photosynthesis of *N. japonicum*, we analyzed several representative JIP test parameters which can reflect the conformational, structural, and functional integrity of photosynthetic apparatus (Figure 10).

Basic fluorescence characteristics showed dose-dependent decreases in Fm and Fv. Fv/Fm was significantly reduced in the 150–200 μM groups, with an inhibition rate of only 3.19% in the 50 μM group. The rise in Fo under high concentrations indicated reversible inactivation or damage to a portion of PSII reaction centers. Fv/Fo showed a trend similar to Fv/Fm and was more sensitive to the elevation of Fo. The inhibition rate at 200 μM was significantly higher than that at 50 μM, confirming that UV-328 suppresses PSII activity in a dose-dependent manner.

Electron transport chain analysis indicated that UV-328 selectively acted on specific sites. Vj, which reflects QA^−^-to-QB electron transfer, increased by 21.54% at 200 μM and 14.38% at 150 μM, but only by 3.92–6.48% at 50–100 μM. This suggests that step is a primary target. Vi, reflecting the transfer from PSII to PSI, showed a smaller change (3.77–8.69%), suggesting higher resistance. Sm and reaction center number (N) showed a biphasic response with slight promotion at 100 μM and inhibition at higher doses. At 150–200 μM, inhibition rates reached 23.17–37.56% for Sm and 29.75–33.41% for N, indicating high concentrations impair reaction center function and reduce their count.

Energy flow analysis showed that ABS/CSm decreased with increasing stress concentration. The inhibition rate was 23.18% at 50 μM and 65.05% at 200 μM, indicating that high-concentration stress damages the light-harvesting capacity of the PSII antenna system. DIo/CSm showed the smallest change, with reductions ranging from 8.70% to 33.54%, suggesting that plants rely on non-photochemical dissipation to alleviate stress damage. The order of impairment in energy transfer efficiency was REo/CSm > ETo/CSm > TRo/CSm. REo/CSm, reflecting NADPH production, was the most significantly inhibited with 88.78% at 200 μM. This indicates that UV-328 primarily blocks the downstream portion of the electron transport chain.

The results showed that photosynthetic performance indices were highly sensitive to UV-328. The total performance index (PI) was inhibited by 74.08% on average. Significant inhibition occurred even at 50 μM, and at 200 μM the inhibition reached 93.51%, indicating nearly complete loss of photosynthetic function. The PI representing PSII alone showed slightly lower inhibition (90.66% at 200 μM), suggesting that the combined action of PSI and PSII is more vulnerable to stress. Threshold analysis revealed that most parameters became significantly inhibited starting at 100–150 μM. Only PI and REo/CSm were already markedly reduced at 50 μM. The number increased to 5 at 100 μM and 12 at 150 μM and above. Thus, the critical threshold for UV-328-induced photosynthetic damage of *N. japonicum* lies between 100 and 150 μM.

## 3. Discussion

### 3.1. Damage on Morphology and Pigment System

UV-328 stress induced dose-dependent morphological damage in *N. japonicum*. Leaf yellowing visibly indicates chlorophyll degradation and is a typical response of both bryophytes and vascular plants to exogenous substance stress [44,45,52,53,54]. Chlorophyll plays an essential role in photosynthesis [55]. Environmental stresses that disrupt chlorophyll metabolism can break the balance between chlorophyll a and b conversion. This disturbance subsequently impairs the efficiency of photon capture and energy transfer [56,57]. These results align with the reductions we observed in chlorophyll content and photosynthetic efficiency.

Leaf surface shrinkage and papillae collapse confirm that UV-328 disrupts the outer protective structures of *N. japonicum* leaves. In bryophytes, which lack thick cuticles and stomatal regulation, papillae are essential to leaf structure and physiological functions. The raised shape of these papillae helps scatter light and reduce photodamage. Also, cell wall polysaccharides and phenolics help trap pollutants and remove ROS. Damage to these structures markedly reduces water retention capacity, making the plants more vulnerable to other stresses [47]. Similar ultrastructural deformation observed in *Leptodictyum riparium* under heavy metal stress confirms the ecological significance of such impairment [58]. Overall, these results suggest that bryophytes can serve as sensitive indicators of UV-328 pollution.

### 3.2. Dose-Dependent ROS Accumulation and Chloroplast Damage

This study confirms that UV-328 stress induces dose-dependent chlorophyll degradation and excessive ROS accumulation in *N. japonicum*, providing direct evidence for the mechanism of UV-328-induced photosynthetic impairment in bryophytes. LSCM observations revealed a clear dose-dependent effect of UV-328 on ROS accumulation and chloroplast integrity. As a sensitive bryophyte without cuticles and stomata, *N. japonicum* captures early cellular stress responses that are often unnoticed in tolerant vascular plants [59]. With rising UV-328 concentration, ROS fluorescence signals gradually intensified, paralleling the progressive loss of chloroplast structural integrity. This pattern suggests that antioxidant defenses [60,61] kick in first but eventually get overwhelmed. The excess ROS then triggers lipid peroxidation in thylakoid membrane [62] and irreversible chloroplast damage through oxidative destruction of photosynthetic components [54]. This matches well with the subsequent observation of dual impairment on both the donor and acceptor sides of PSII.

### 3.3. Dose-Dependent Impairment of PSII Function

Analysis of chlorophyll fluorescence parameters and OJIP kinetic curves helped clarify how UV-328 damages the photosynthetic apparatus. A drop in Fv/Fm typically signals initial injury to PSII reaction centers [53]. However, the decrease in Fv/Fm was smaller than that in Y(II), suggesting that the damage starts from the reaction center structure and then extends to electron transport. Our observation of elevated Vj confirms this pattern. So, it appears that UV-328 first inflicts structural harm on PSII reaction centers. This leads to a modest reduction in maximum photochemical efficiency, which in turn hinders electron transport under actual photosynthetic conditions. At the molecular level, the damage likely involves oxidative attack on core PSII proteins like D1. There may also be links to changes in the expression of genes responsible for light-harvesting complexes and UV-B perception [28].

A marked drop in qP was observed, pointing to a decline in photochemical reaction intensity [63]. The sharp decreases in both qP and Y(II) suggest that UV-328 damages PSII reaction centers and suppresses photochemical energy conversion by blocking electron transport from QA to QB [34]. In contrast, qN increased slightly, indicating that *N. japonicum* has a limited thermal dissipation capacity under UV-328 stress. This response reflects the evolutionary traits of bryophytes. Unlike vascular plants, bryophytes lack an efficient non-photochemical quenching system that depends on the xanthophyll cycle. Instead, their thermal dissipation relies primarily on physical relaxation of thylakoid membranes or chemical quenching by small amounts of phenols, which cannot be effectively activated under severe stress.

### 3.4. Multi-Target Damage to the Photosynthetic Electron Transport Chain

Fast chlorophyll fluorescence induction kinetics acts as a molecular probe for primary PSII photochemistry. Its polyphasic rise allows us to locate damage sites with reasonable precision [64,65]. OJIP curve analysis identified specific UV-328 action sites in *N. japonicum*. Under high-concentration stress, the polyphasic transient almost disappeared, indicating a total breakdown of photosynthetic function. The observed increase in J-phase fluorescence confirms that electron transfer from QA^−^ to QB on the acceptor side is blocked, which is fully consistent with the 21.54% elevation of Vj at 200 μM. Meanwhile, the appearance of K-phase is a well-known sign that OEC on the donor side has been impaired [48]. Because OEC serves as the core unit for water splitting and initial electron supply, its inactivation directly cuts off the electron flow from water molecules to the PSII reaction center. As a result, the donor side receives too few electrons, giving rise to a clear K-band [66,67].

Mechanistically, the OEC comprises a Mn_4_CaO_5_ cluster and three extrinsic protein subunits that stabilize the catalytic center on the lumenal side of PSII. Given the high hydrophobicity of UV-328, we propose that it may integrate into the thylakoid membrane, thereby disrupting the local lipid environment and interfering with the conformational stability of these OEC subunits. Such disruption would impair the efficient extraction of electrons from water, leading to a donor-side bottleneck that manifests as the observed K-band appearance and the subsequent decline in electron transport parameters. While this interpretation provides a plausible mechanistic linkage, we acknowledge that these are speculations drawn from our fluorescence data and remain to be substantiated by direct biochemical evidence. This represents a significant direction for our future investigations.

L-band analysis indicated that energy connectivity among PSII units was reduced, and the mechanism for sharing excitation energy became disrupted [53,65]. This loss of connectivity seems closely tied to OEC damage. OEC inactivation interrupts donor-side electron flow, trapping excessive excitation energy in the antenna system and further aggravating photo-oxidative damage [68]. Similar patterns have been reported in studies on heat stress in *Ageratina adenophora.* As stress intensified, L-band intensity rose while OEC activity declined, with both forms of damage becoming more severe [53]. These observations align well with what we saw in our experiments.

The kinetic analysis of the O-I phase offers additional insight into how photosynthetic damage transmits hierarchically. Under low UV-328 stress, negative ΔW_OI_ values suggest a compensatory activation of PSI, which accelerates the re-oxidation of the PQ pool. In contrast, ΔW_OI_ remains positive under 200 μM stress, indicating that the stress has surpassed PSI’s compensatory capacity, leading to over-reduction in the PQ pool [69,70]. All treatment groups show negative ΔW_OI_ values in the I-P phase, but at 200 μM the negative deflection is markedly smaller. This points to over-reduction in the terminal acceptors of PSI and a consequent block in NADPH production [71]. These observations are fully consistent with the 88.78% inhibition rate of REo/CSm detected in this study.

It is worth noting that the sensitivity to UV-328 differs considerably among plant species. In bryophytes like *N. japonicum*, the absence of a well-developed cuticle, combined with a high surface-to-volume ratio and strong cation exchange capacity, facilitates direct and rapid pollutant absorption. This structural and physiological predisposition likely accounts for the higher susceptibility of bryophytes compared to vascular plants. By contrast, *Arabidopsis thaliana* exhibits moderate sensitivity, with reported effects at 50 μM UV-328 that involve disruption of mineral nutrient homeostasis, reprogramming of secondary metabolism, and activation of antioxidant enzyme genes [14]. Rice shows comparable moderate sensitivity, where UV-328 suppresses chlorophyll synthesis genes by over 50% while activating chlorophyll degradation pathways, accompanied by broader metabolic reprogramming of carbohydrate and amino acid metabolism [33]. In algae, toxicity appears to be driven primarily by bioaccumulation, which disrupts photosynthetic electron transport and induces oxidative overload, often with membrane-associated disruption [35]. Collectively, these comparisons underscore that the high sensitivity of bryophytes to UV-328 stems from a combination of their unique morphological and physiological traits, making them particularly suitable as sentinel organisms for monitoring emerging organic pollutants in terrestrial ecosystems.

### 3.5. Energy Allocation Imbalance and Reaction Center Inactivation

Parameters related to specific energy fluxes in active PSII reaction centers (RCs) reveal how light energy is partitioned among individual functional RCs [64,72]. ABS/RC increased with rising UV-328 concentration, indicating a reduction in active RCs numbers and a higher light-harvesting load imposed on the remaining functional centers. TRo/RC remained stable while ETo/RC decreased significantly, showing a clear uncoupling between light capture and downstream electron transfer. This suggests that PSII RCs still retain the capacity for light absorption and excitation trapping, but the downstream electron transport chain is severely impaired [71].

As an index of non-photochemical energy dissipation per functional RC, DI_0_/RC largely relies on NPQ pathways to alleviate photooxidative damage arising from excessive excitation of reaction centers [65,73]. The sharp increase in DIo/RC, particularly at 200 μM, reflects preferential activation of the thermal dissipation pathway, forming a survival-oriented energy allocation strategy. A similar pattern has been reported in *N. japonicum* exposed to strontium stress, where enhanced NPQ helped vent excess light energy as heat [44].

## 4. Conclusions

In this study, we systematically investigated the phytotoxic effects of UV-328 on the photosynthetic apparatus of *Niphotrichum japonicum*, a representative bryophyte species in East Asia. Our findings demonstrate that UV-328 induces dose-dependent, multi-target damage to the PSII photosynthetic system, with a critical damage threshold identified at 100–150 μM.

At the morphological and physiological levels, UV-328 exposure caused leaf yellowing, papillae collapse, and significant chlorophyll degradation, accompanied by dose-dependent ROS accumulation and progressive chloroplast disruption. Chlorophyll fluorescence imaging and OJIP transient analyses further revealed that UV-328 simultaneously impairs both the donor and acceptor sides of PSII. Specifically, the OEC is damaged, as evidenced by the emergence of a K-band, while electron transfer from QA^−^ to QB is blocked, as indicated by elevated J-phase fluorescence. In addition, PSI terminal acceptor reduction is inhibited, leading to a bottleneck in NADPH production. Collectively, these impairments decouple light energy capture from downstream electron utilization, forcing an energy allocation shift toward thermal dissipation and ultimately causing a catastrophic decline in overall photosynthetic performance at 200 μM.

Our results provide the first systematic evidence that UV-328, as an emerging persistent organic pollutant, disrupts bryophyte photosynthesis through a multi-tiered mechanism. The identification of a specific toxicity threshold, combined with the extreme sensitivity of bryophytes to this compound, highlights the potential utility of *N. japonicum* as a sentinel organism for monitoring UV-328 contamination in terrestrial ecosystems. Furthermore, the dose-dependent response patterns and the relatively stable photosynthetic performance below 100 μM suggest that bryophytes may possess a certain capacity to tolerate and accumulate UV-328 without suffering catastrophic damage, opening up new research avenues into their potential role in phytoremediation of polluted environments. Given the recent global regulation of UV-328 and its widespread environmental occurrence, these findings contribute to a more comprehensive understanding of the ecological risks posed by this pollutant to non-vascular plants and terrestrial ecosystems.

Admittedly, the mechanistic speculation regarding the disruption of the OEC requires more direct biochemical validation. Employing transcriptomic and proteomic approaches to elucidate the molecular pathways underlying UV-328-induced photosynthetic damage represents a key priority for our future investigations. These efforts will not only refine our understanding of the toxic mechanisms but also facilitate the development of effective biomonitoring and remediation strategies for UV-328-contaminated environments.

## 5. Materials and Methods

### 5.1. Plant Materials and Treatments

The moss (*N. japonicum*) used in this study was collected from Lishui, Zhejiang, China. Before being carefully rinsed with deionized water to remove the stone fragments and ruderal plants, the *N. japonicum* mats were cut into 10 cm × 10 cm sizes and planted in plastic boxes. These boxes were then placed in a climatic chamber with the growth conditions maintained at a 16 h/8 h light–dark cycle, 25 °C/20 °C day/night temperature, 65% humidity, and 40 μmol m^−2^ s^−1^ photosynthetic photon flux density for 2 days before stressed by UV-328 (Aladdin, Shanghai, China) treatment. UV-328 was dissolved in DMSO (Aladdin, Shanghai, China) to obtain a 100 mM stock solution and added to autoclaved BCD medium (Ybio, Shanghai, China) at the specified concentrations. According to the previous reports of UV-328 stress on *Arabidopsis thaliana* [28], we tested a series of concentrations in preliminary experiments and observed that moss growth was clearly suppressed at 200 µM UV-328. Therefore, concentrations of 0, 50, 100, 150, and 200 µM were selected for this study. Moss mats were immersed in a plastic box with 25 mL UV-328 solutions which were replaced every day. Each treatment includes 3 boxes of moss mats. After 5 days of cultivation, samples were randomly collected to measure various biological and photosynthetic parameters. Each parameter was tested in at least three replicates per experiment, and the data shown are the averages of three separate biological experiments.

### 5.2. Morphological and Structural Analysis

After 5 days treatment, samples were randomly selected and surface-rinsed with deionized water for photography. Three samples were imaged with stereomicroscope (SZ61, Olympus, Tokyo, Japan). Meanwhile, 5 g of fresh moss samples were randomly weighed, washed, dried to constant weight at 70 °C, ground with liquid nitrogen, and then subjected to microstructure photography using a scanning electron microscope (SU8600, HITACHI, Hitachi, Japan).

### 5.3. ROS Localization

*Niphotrichum japonicum* plants were harvested after 5 days of UV-328 exposure for the estimation of ROS production [74]. Fresh gametophyte samples were incubated with 25 µM DCF-DA (Aladdin, Shanghai, China), a widely used fluorescent probe for visualizing ROS accumulation, for 10 min and then washed three times in 10 mM Tris-HCl (pH 7.4) (Aladdin, Shanghai, China) for 30 min. Leaves were then examined under a confocal laser scanning microscope (LSM900, Zeiss, Oberkochen, Germany). Excitation of DCF-DA and chlorophyll was set at 405 nm and 496 nm wavelength, and emission bandwidths were detected at 506–585 nm (green light) and 615–715 nm (red light), respectively. Observations were repeated 3 times for each treatment.

### 5.4. Chlorophyll Measurement and Fluorescence Imaging

Chlorophyll of *N. japonicum* gametophytes (0.3 g fresh weight) was extracted with 10 mL of acetone-ethanol mixture with 1:1 (*v*/*v*) at room temperature in the dark for 24 h until complete chlorosis was observed. The chlorophyll content was determined by absorbances at 663 nm and 645 nm according to the method described previously [46] using a multi-mode microplate reader (Synergy H1, Agilent BioTek, Hayward, CA, USA). Chlorophyll fluorescence parameters and images were obtained as described previously [45]. After 5 days of exposure to various UV-328 concentrations, *N. japonicum* gametophytes were collected and dark-adapted for 20 min. Fluorescence was measured using a chlorophyll fluorescence imaging system (Imaging-PAM MAXI, WALZ, Munich, Germany).

### 5.5. Chlorophyll a Fluorescence Induction Curve Measurement and JIP Test

After being dark-adapted for 30 min, the OJIP curve of *N. japonicum* was determined using Handy PEA fluorometer (Handy PEA, Hansatech, King’s Lynn, UK) [64,75,76]. The OJIP curves were elicited by a 1 s pulse of red light at 3000 μmol photons m^−2^ s^−1^. Fluorescence intensities at specific times are defined as F_O_ at 20 μs, F_L_ at 150 μs (L-step), F_K_ at 300 μs (K-step), F_J_ at 2 ms (J-step), and F_I_ at 30 ms (I-step). The maximal fluorescence intensity (F_M_) corresponds to F_P_ measured under saturating light. V_t_, the relative variable fluorescence at time t, is defined as V_t_ = (F_t_ − F_O_)/(F_M_ − F_O_) for O-P normalization. To reveal the L-band, W_OK_ = (F_t_ − F_O_)/(F_K_ − F_O_) is used for O-K standardization. Similarly, the K-band was displayed using W_OJ_ = (F_t_ − F_O_)/(F_J_ − F_O_) (O-J standardization), and the J-step using W_OI_ = (F_t_ − F_O_)/(F_I_ − F_O_) [77]. The relative variable fluorescence at the L- and K-steps are given by W_L_ = (F_L_ − F_O_)/(F_J_ − F_O_) and W_K_ = (F_K_ − F_O_)/(F_J_ − F_O_), respectively [78].

The JIP test was used for data analysis [64,79]. This test characterizes the maximal energy fluxes along the photosynthetic chain, including absorption (ABS), trapping (TRo), electron transport (ETo), energy dissipation (DIo), and the excited leaf cross-section (CS). The maximum quantum yield of primary photochemistry in PSII is defined as TRo/ABS = 1 − (F_O_/F_M_). The probability of electron transfer beyond QA^−^ was calculated as ETo/TRo = 1 − Vj. Overall PSII performance was assessed using the absorption-based performance index (PI_abs_ or PI_total_).

### 5.6. Statistical Analysis

One-way ANOVA with Tukey’s post hoc test (*p* < 0.05) was applied to evaluate statistical significance using SPSS v19.0. Results are presented as means ± SD of independent replicates. OriginLab software (version Origin 2021) was used for basic data visualization and processing, and the number of replicates is indicated in the figure legends.

## Data Availability

Data available on request from the corresponding author. The data are not publicly available due to privacy.
